# Interprofessional collaboration within general practice teams following the inclusion of non-dispensing pharmacists

**DOI:** 10.1186/s40545-023-00550-3

**Published:** 2023-03-21

**Authors:** Thilini Sudeshika, Louise S. Deeks, Mark Naunton, Gregory M. Peterson, Sam Kosari

**Affiliations:** 1 Discipline of Pharmacy, Faculty of Health, University of Canberra, Bruce, ACT 2617 Australia; 2 Department of Pharmacy, Faculty of Allied Health Sciences, University of Peradeniya, Peradeniya, 20400 Sri Lanka; 3 School of Pharmacy and Pharmacology, University of Tasmania, Hobart, TAS 7005 Australia

**Keywords:** General practice pharmacist, Interprofessional collaboration, Team effectiveness, Trust, Professional working relationships, Barriers

## Abstract

**Background:**

Pharmacists have been included in general practice teams to provide non-dispensing services in the Australian Capital Territory (ACT) since 2016. Interprofessional collaboration and team effectiveness are key considerations in providing high-quality patient care. These concepts have not been well studied following the inclusion of a pharmacist in general practice teams.

**Methods:**

A mixed methods study was conducted to explore collaboration between pharmacists and health professionals in eight general practices in the ACT, where pharmacists were included in their teams. A validated survey instrument was adapted and utilised to assess the changes in interprofessional collaboration over time following the addition of a pharmacist. Another validated survey was utilised to explore team effectiveness at the end of the study. Semi-structured interviews, with a thematic analysis, were conducted with a purposeful sample of general practice staff members to understand the factors influencing the development of interprofessional collaboration.

**Results:**

In total, 56 and 41 participants completed the baseline and follow-up survey, including 26 who completed both surveys to assess the change in collaboration over time. Interprofessional collaboration scores were high initially and did not change over time. Team effectiveness was also high at the end of the study. Twenty-one individuals participated in interviews, which generated four main interrelated themes related to interprofessional collaboration: professional working relationships, trust, commitment to collaboration, and barriers to collaboration. Trust was integral to professional working relationships and commitment to collaboration. The barriers to collaboration included not having a role description for pharmacists, inadequate interest to initiate working relationships, lack of dedicated time for interaction, lack of utilisation, and poor awareness of pharmacist-led activities in general practice.

**Conclusion:**

Interprofessional collaboration was initially high and not influenced by the addition of a pharmacist, perhaps reflecting the inherent nature of the general practices willing to include a pharmacist within their team. Introducing a clear job description for pharmacists, and dedicating time to interact with pharmacists, could be beneficial in improving trust and professional working relationships and enhancing collaboration between the pharmacists and other general practice team members.

**Supplementary Information:**

The online version contains supplementary material available at 10.1186/s40545-023-00550-3.

## Introduction

As pharmacists’ roles have expanded to deliver more comprehensive patient care in healthcare teams, non-dispensing pharmacists have been employed in general practices in many countries [1–5]. Their main purpose is to support general practitioners (GPs) in reducing medication-related risks and optimising medication use. Studies have shown that pharmacist-led services can benefit patients and general practice teams through numerous activities, such as providing education to patients and staff, undertaking medication reviews, conducting clinical audits, updating medical records, and administering vaccines [1–5]. This role requires effective collaboration with GPs and other health professionals. Despite this need for collaboration, little is known about the factors related to effective collaboration between the pharmacist and other general practice staff members [1, 5–7].

Interprofessional collaboration is considered a key factor in successfully implementing team-based care models. Interprofessional collaboration is defined by the World Health Organization (WHO) as “people from different disciplines working together with patients, families, caregivers, and communities to deliver the highest quality of care” [8]. Interprofessional collaboration in healthcare not only helps to improve patient safety and outcomes, but also helps to reduce inefficiencies and costs [9, 10]. Moreover, it has been reported that interprofessional collaboration improves the collective awareness of health professionals’ knowledge and skills, contributing to quality of care through continued improvement in decision-making [11]. In contrast, a lack of collaboration between health professionals can lead to poor outcomes, dissatisfaction and harmful consequences for patients [12]. The International Pharmaceutical Federation and WHO have highlighted the significance of pharmacists’ collaborative activities in team-based care models [13]. Understanding the factors impacting interprofessional collaboration is required to optimise high-quality care for patients.

Employing pharmacists in general practice teams is gradually increasing across Australia [14]. In the Australian Capital Territory (ACT), pharmacists’ services were first introduced in general practices in 2016 [15]. After promising results in a pilot study, pharmacists’ services in general practices expanded further through funding from the Capital Health Network (CHN: ACT’s primary healthcare network). As the general practice pharmacist’s role is relatively new in Australia, the collaboration between the pharmacist and other general practice team members may be challenging and is not well understood. This study aimed to assess the changes in interprofessional collaboration following the introduction of pharmacists in general practice teams; understand the factors impacting the development of interprofessional collaboration between the pharmacist and other general practice team members; and assess the level of team effectiveness in general practice when a pharmacist was within the general practice teams.

## Methods

### Design

**Fig. 1:**
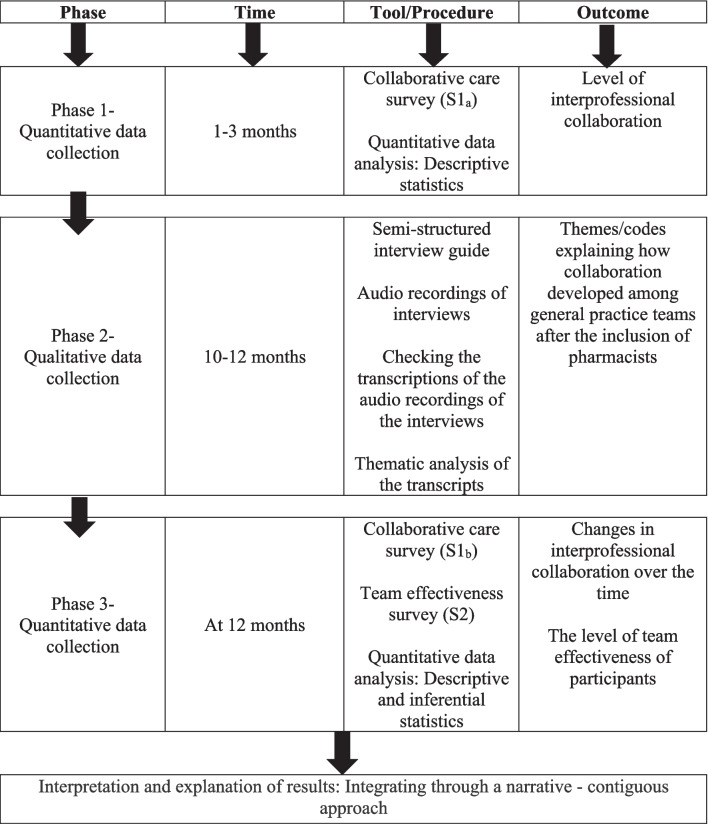
Mixed methods integration flow diagram

This study utilised a mixed methods design to understand the holistic view of interprofessional collaboration and team effectiveness of general practice teams following the inclusion of pharmacists. A multiphase sequential explanatory design was utilised. In the first phase, a baseline survey (S1_a_) was conducted to investigate the interprofessional collaboration of general practice team members (Fig. 1). This was followed by the conduct of in-depth interviews in the second phase with general practice pharmacists, GPs, and other health professionals to gain insight into the factors impacting the development of interprofessional collaboration after including a pharmacist in the general practice team. In the third phase, the same survey (S1_b_) as in the initial stage was utilised to assess the changes of interprofessional collaboration over time. An additional survey (S2) was administered in the third phase to explore the team effectiveness of general practice teams after including pharmacists. The protocol for this study has been published [16]. The researchers were not involved in the recruitment of general practices or employment of pharmacists. Ethical approval for the study was obtained from the human research and ethics committee at the University of Canberra (HREC 15–235).

### Study instruments

#### Collaborative care and team effectiveness surveys

Collaborative care and team effectiveness surveys were adapted from previously validated tools [17–20]. The collaborative care survey included demographics, professional interaction exchange characteristics (relationship initiation, trust, and role clarity), and commitment to collaboration (Additional file 1). The statements within professional interactions were adapted from the Pharmacist Frequency of Interprofessional Collaboration Instrument (FICI-P) and the Physician–Pharmacist Collaborative Index (PPCI) [17, 20]; exchange characteristics (relationship initiation, trust, and role specification) were modified from the PPCI [17], and commitment to collaboration was extracted from an extension to the PPCI [18].

The survey for pharmacists and GPs had the same number of items (total items *n* = 21: professional interactions *n* = 4, relationship initiation *n* = 3, trust and role clarity *n* = 10, commitment to collaboration *n* = 4). The survey for other health professionals had fewer items due to the inapplicability of some statements in the validated tools (total items *n* = 17: professional interactions *n* = 4, relationship initiation *n* = 3, trust and role clarity *n* = 8, commitment to collaboration *n* = 2). Total collaboration scores were calculated in the first and third phases [18, 21].

A 24-item questionnaire on team effectiveness was adapted from the Primary Care Team Dynamics Survey (Additional file 2) [19]. The respondents were asked to indicate the frequency of professional interactions on a 1 to 4-point scale, and their extent of agreement or disagreement with the statements in other domains on a 1 to 5-point scale. The face validity was reviewed and the survey was pre-tested by experts, as described previously [16]. Total scores for team effectiveness were calculated at 12 months.

#### Semi-structured interviews

The semi-structured interview guide was developed to gain an understanding of the views of individuals on three main domains—role clarity, professional interactions, and collaboration (Additional file 3) [16, 22]. This guide was designed by considering previous studies and was pre-tested with a general practice pharmacist.

### Setting and intervention

The study was conducted in eight general practices in the ACT, Australia where the general practice pharmacist model was being trialled. Pharmacists were employed in these general practices on a part-time basis (15 h per week) to provide non-dispensing services for patients and work with GPs and other health professionals [16].

### Participants and data collection

This study targeted all pharmacists, GPs, GP registrars (fully qualified medical doctors, undertaking advanced training to specialise in general practice), and other health professionals (e.g. nurses, nurse practitioners, psychologists, and physiotherapists) in the recruited general practices (*n* ≈ 113) between June 2019 and April 2021 [16]. Pharmacists were invited to participate in an online survey and other participants were invited to complete a paper-based survey. The surveys were distributed to all participants at two timepoints (Fig. 1) and were open for 4–8 weeks. To preserve confidentiality, the paper-based responses were collected in locked boxes, which only the primary researcher (TS) could access.

For the interviews, participants were approached individually to discuss their participation by TS, following a letter of invitation. Written consent was obtained from all participants prior to the interviews. A maximum variation sampling technique was utilised to recruit participants aiming to reflect the widest range of views from a heterogeneous sample. This included recruiting at least one pharmacist, one GP, and one other health professional from each study site. Interviews were conducted by an experienced qualitative interviewer (LSD) via telephone, and written notes were taken during the interview. Full interviews were audiotaped, de-identified, and transcribed verbatim by an independent professional transcribing service.

### Data analysis

Descriptive statistics was performed to summarise the demographic details of the participants. Paired t-tests were performed to assess the differences between scores for professional interactions, relationship initiation, exchange characteristics (trust and role clarity), commitment to collaboration and overall interprofessional collaboration over time [16]. A *p*-value < 0.05 was considered statistically significant. The data were analysed using Statistical Package for the Social Sciences (SPSS ver. 27 IBM Corp, Armonk, New York, USA).

Thematic analysis was utilised to analyse the qualitative data [23]. Interview data were coded and analysed independently by two investigators (TS, LSD). Discrepancies of codes were resolved by another researcher (SK). The emerging themes and sub-themes were reviewed and finalised with the research team. Thematic analysis was performed with the assistance of NVivo qualitative data analysis software (NVivo ver. 12, QSR, Melbourne, VIC, Australia).

## Results

### Changes in interprofessional collaboration over time

In the first phase, 56 participants from eight general practices completed the survey (response rate approximately 50%) including pharmacists (*n* = 8), GPs (*n* = 31), and other health professionals (*n* = 17). Forty-one participants completed the survey (response rate 40%) in the third phase, including 26 participants (Table 1) who had completed the initial survey and therefore paired data had been provided (Table 2).

**Table 1:** Demographics of the participants

Phase	Demographic	Pharmacists	GPs/GP registrars	Other health professionals
Phase 1 and 3: Survey (Changes in collaboration over time)	Participants (n = 26)	7	16	3
	Age (years) (n, %)			
	20–30	1 (14)	1 (6)	0
	31–40	3 (43)	6 (38)	0
	41–50	1 (14)	3 (19)	2 (67)
	More than 50	2 (29)	6 (38)	1 (33)
	Gender (n, %)			
	Male	4 (57)	5 (31)	0
	Female	3 (43)	11 (69)	3 (100)
	Experience as a registered healthcare professional (years) (n, %)	1 (14)	0	0
	Less than 5	2 (29)	6 (38)	0
	5–11	2 (29)	2 (13)	1 (33)
	12–18	0	5 (31)	1 (33)
	19–25	2 (29)	3 (19)	1 (33)
	More than 25	3 (43)	N/A	N/A
	Accreditation status^*^ (n, %)			
	Prior working background (n, %)			
	Community pharmacy	4 (57)	N/A	N/A
	Hospital pharmacy	2 (29)		
	Other	1 (14)		
Phase 2: Interviews (Factors impacting collaboration)	Participants (n = 21)	7	6	8
	Gender (n, %)			
	Male	4 (57)	3 (50)	1 (12)
	Female	3 (43)	3 (50)	7 (88)
Phase 3: Survey (Team effectiveness)	Participants (n = 41)	7	29	5
	Age (years) (n, %)			
	20–30	1 (14)	2 (7)	0
	31–40	3 (43)	10 (34)	0
	41–50	1 (14)	7 (24)	2 (40)
	More than 50	2 (29)	10 (34)	2 (40)
	Not answered	0	0	1 (20)
	Gender (n, %)			
	Male	4 (57)	13 (45)	1 (20)
	Female	3 (43)	16 (55)	3 (60)
	Not answered	0	0	1 (20)
	Experience as a registered healthcare professional (years) (n, %)			
	Less than 5	1 (14)	1 (3)	1 (20)
	5–11	2 (29)	11 (38)	0
	12–18	2 (29)	4 (14)	1 (20)
	19–25	0	6 (21)	1 (20)
	More than 25	2 (29)	7 (24)	2 (40)

Due to rounding, percentages may not always add up to 100%

*Registered pharmacists who have accreditation to perform medication management reviews

**Table 2:** The changes in interprofessional collaboration survey scores over time

Domain (maximum score)	Pharmacists *n* = 7			GPs n = 16		
	Phase 1- S1_a_ (Mean ± SD)	Phase 3—S1_b_ (Mean ± SD)	*p-*value	Phase 1—S1_a_ (Mean ± SD)	Phase 3—S1_b_ (Mean ± SD)	*p-*value
Professional interactions (/20)	15.1 ± 3.0	15.6 ± 3.1	0.79	11.8 ± 4.2	11.7 ± 5.5	0.96
Relationship initiation (/15)	12.7 ± 2.4	14.0 ± 1.5	0.08	10.3 ± 2.9	9.8 ± 3.4	0.65
Trust and role specification (/50)	43.3 ± 6.6	43.0 ± 5.4	0.93	44.4 ± 4.7	43.2 ± 5.3	0.48
Commitment to collaboration (/20)	17.9 ± 2.0	17.0 ± 2.1	0.58	16.3 ± 2.8	15.9 ± 3.3	0.71
Total score (/105)	89.0 ± 10.7	89.6 ± 9.2	0.92	82.7 ± 11.1	80.6 ± 14.9	0.62

S1_a_ Baseline collaborative care survey

S1_b_ Folllow-up survey

Due to the low follow-up response rate (18%), other health professionals’ scores (*n* = 3) were excluded from this analysis. Therefore, 23 paired responses from pharmacists and GPs were included to assess the changes in interprofessional collaboration over time. The scores were relatively high at baseline, and neither pharmacists’ nor GPs’ survey scores changed significantly over the study period (Table 2). Pharmacists appeared to rate higher scores for relationship initiation than GPs in the third phase (*p* < 0.05), while pharmacists and GPs rated similar scores for the other domains at both timepoints.

### Factors impacting the development of interprofessional collaboration

**Fig. 2:**
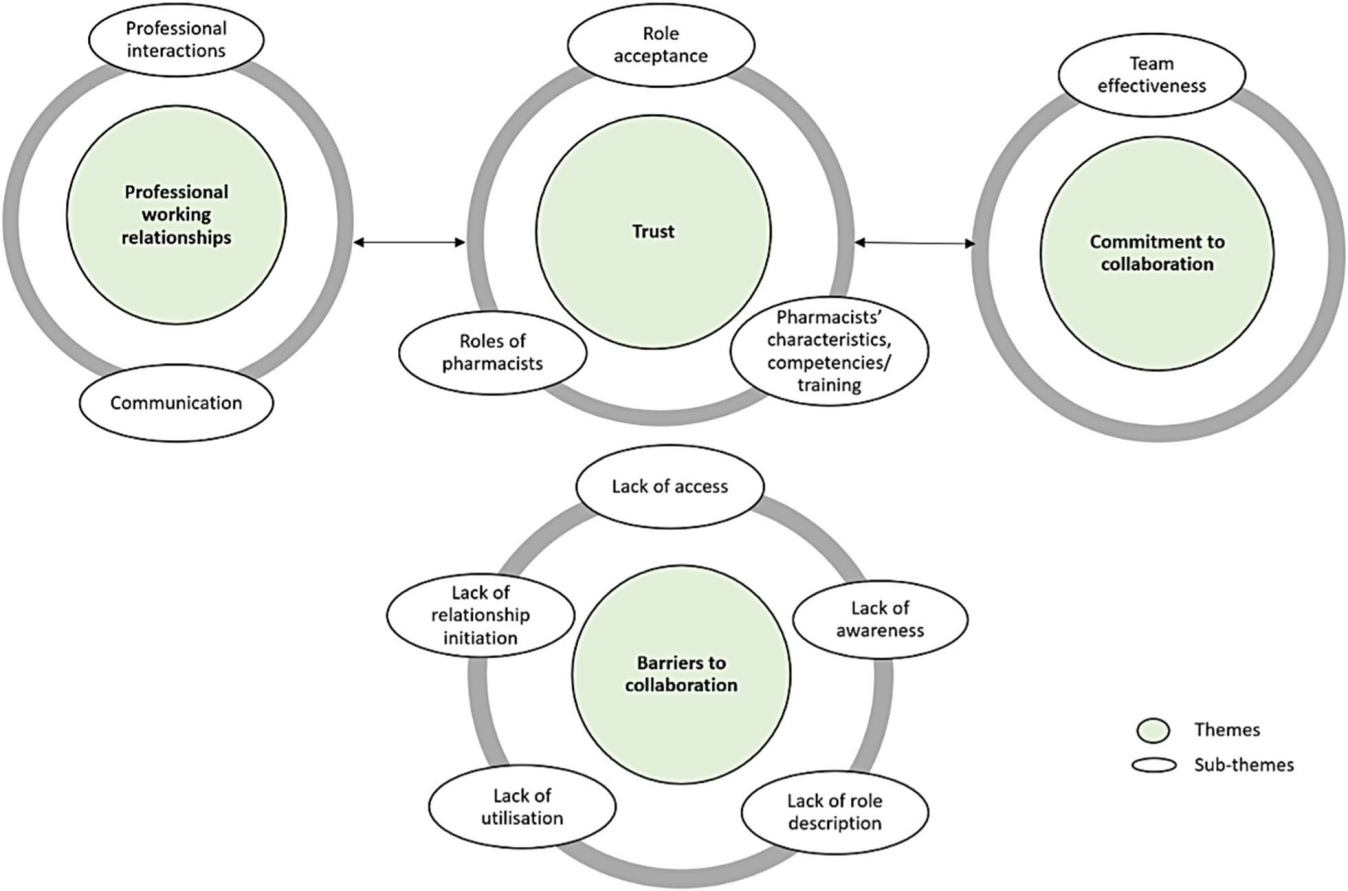
Factors influencing the development of collaboration between pharmacists and other general practice team members

In the second phase, 21 participants (Table 1) were interviewed to gather in-depth details of collaboration between the pharmacist and general practice team members. This sample included 7 pharmacists, 5 GPs, one GP registrar, 6 nurses, one psychologist, and one alcohol misuse counsellor. The thematic analysis generated four predominant interrelated themes: professional working relationships, trust, commitment to collaboration, and barriers to collaboration (Fig. 2). Illustrative data relevant to the themes and sub-themes are italicised in the following text.

#### Professional working relationships

Professional interactions between the general practice pharmacists and other general practice team members occurred through medication-related queries, formal/informal meetings, case conferences, health assessments, care plans, and referrals for medication reviews or providing education to patients. Participants emphasised the importance of formal or informal meetings to improve their working relationships and communication with each other. Most informal meetings occurred over lunch or coffee time which facilitated more members of the general practice team to be involved in discussions.

*“We try to really meet with the pharmacist maybe once a fortnight or once a month to really see what are the things that the pharmacist has suggested, or identified, what did they think the issues are. But very often we use the case conference as opportunity almost like a talking through patient's care and using the same time to let the pharmacist to provide their feedback in terms of what they have identified so far.”* GP 3

*“I guess in this practice we're lucky that we—all the doctors and staff have lunch at the same time, and we have a lovely luncheon where we often just all sit and talk. Sometimes it's just what did you do on the weekend. Sometimes it was case conferences about patients that had been seen during the week or during the day that people needed more input on.”* Nurse 5

Furthermore, pharmacists acted as a conduit between the general practice and external services, such as community pharmacies, hospitals, and aged care facilities. Pharmacists discussed their professional interactions with the community pharmacists and external health services in their area.

Participants expressed their views on the frequency and modes of communication. Internal messenger systems, emails, face-to-face conversations, and telephone calls were the modes of communication between the general practice pharmacist and other general practice team members. Most participants believed that they had open communication in their general practices.

*“In our two roles, XXXX and I communicate quite frequently. Sometimes it’s even more, depending on what we’re working on.”* Nurse 6

Almost all GPs and other health professional interviewees said that general practice pharmacists were easy to approach; however, a barrier to communication was the part-time hours of health professionals in general practice teams that limited working relationships. Pharmacists discussed the strategies that they utilised to increase communication and professional interactions with the GPs to improve professional working relationships.

*“Then I would send them a message to say, oh, I just left such-and-such, a printout of your patients that may benefit from seeing me in your pigeonhole. Can you get back to me in three weeks' time?”* Pharmacist 5

#### Trust

In discussing the trust between the pharmacist and other general practice team members, interviewees highlighted the acceptance or refusal of recommendations, role acceptance, competencies and roles of pharmacists, professional working relationships, and commitment to collaboration. Participants stated that pharmacists’ recommendations were accepted to a considerable extent. They further discussed the reasons for acceptance or refusal of pharmacists’ recommendations.

*“They've been very responsive to my ideas. They listen to what I have to say. It's not that they necessarily implement absolutely everything, but they'll listen to my views and when I say implement everything, there might be a case where they decide to implement it slightly later down the line.”* Pharmacist 4

*“I think, in some ways, pharmacists are extremely trusted, and he’ll be trusted to talk about what the patient’s experience with medication is in some ways more than us as GPs, who are seen as prescribing it, but not necessarily following on with side effects and downsides.”* GP 6

Pharmacists described some situations where they had been frustrated because they felt that GPs had not considered their well-researched clinical recommendations. However, most GPs and nurses emphasised that the pharmacists’ skillset and knowledge base were beneficial to general practices. The general practice team members commented on how pharmacists’ competencies and role clarity improved their trust. Furthermore, participants expressed that trust is a key factor for fruitful collaboration and professional working relationships.

*“She has a skill set that GPs don’t have and we all value that in terms of her pharmacological knowledge and as a XXXX she was extremely valuable to the GPs and practice nurses and patients here that definitely trusted her advice and knowledge.”* GP 2

*“Not always, but mostly, because she is talking from a pharmacist capacity and I'm not a pharmacist, so I very much respect her skill set and training level.”* Nurse 2

#### Commitment to collaboration

Participants described the impact of collaboration and team effectiveness on patient care. Pharmacist-GP-nurse collaboration to identify medication-related problems was reported as an important mechanism to improve integrated care and team effectiveness and reduce workload. Furthermore, participants described that commitment to collaboration evolved over time. Most participants commented that the collaboration between general practice team members could improve the efficiency of the team in patient care. Participants described how making decisions for patients as a team could improve the quality of care in the general practice setting.

*“Also, to get those other member's input on client care or on the best way forward for some of our complex clients. I think that's really vital.”* Nurse 3

*“I think having that collaborative approach and being able to do that, particularly with the pharmacist there, it's made our work much more effective and easier.”* Other Health Professional 2

*“Having everybody involved has the best results. But the more professionals you have, it seems like the better care and the outcomes for the patients are over time.”* Pharmacist 3

#### Barriers to collaboration

Participants identified five major barriers to collaboration (Fig. 2). They commented on the importance of having a role description for pharmacists to improve professional working relationships. Furthermore, lack of awareness of what pharmacists could do in general practice limited the collaboration with pharmacists. Lack of dedicated time for professional interactions between health professionals had restricted the access to collaboration with pharmacists. Moreover, most pharmacists described that part-time working hours limited relationship initiation and collaboration with GPs and other health professionals.

*“I mean, maybe the change here, just thinking about it, needs to be, maybe I need to remember to use the pharmacist more often.”* GP 1

*“Everyone knows what a nurse does, but the pharmacist is a bit of a blank slate.”* GP 4

*“If they have time because a lot of the time, even if I want to talk to them, they don't have time. Yeah, doctors are very busy here because they basically talk to the patients like back-to-back pretty much.”* Pharmacist 7

### Team effectiveness when a pharmacist was within general practice teams

Forty-one participants completed the team effectiveness survey in the third phase (Table 1). As with interprofessional collaboration, the scores were high: 99.8 ± 8.3 for GPs, 100.3 ± 12.8 for pharmacists, and 107.4 ± 9.9 for other health professionals (maximum survey score was 120; mean ± SD, where higher scores represent greater team effectiveness).

## Discussion

This multiphase mixed methods study assessed the collaboration and team effectiveness of health professionals in general practice after including a pharmacist in their teams. Moreover, the study identified factors influencing the development of interprofessional collaboration between pharmacists and general practice team members.

The findings showed that participants’ interprofessional collaboration did not change over the study. The health professionals in the general practice teams already had a high level of collaboration in the early stages of the pharmacists’ employment in general practice and it was maintained over time. This may reflect the willingness of the participating study sites to include a pharmacist within the practice; that is, the participating general practice staff already had high interprofessional collaboration and could probably recognise the benefit of adding a pharmacist to their team. Willingness to collaborate has been identified as a facilitator to introduce pharmacists in general practice teams [5]. High team effectiveness scores suggested that general practice team performance was probably high initially and not greatly affected when a pharmacist was added to their teams.

This study identified four themes related to interprofessional collaboration: professional working relationships, trust, commitment to collaboration, and barriers to collaboration. Interviews revealed that trust was integral to professional working relationships and commitment to collaboration between pharmacists and general practice team members. The findings indicated that professional working relationships between pharmacists and health professionals in general practice teams was influenced by communication and professional interactions. This is consistent with studies that have recognised professional interactions and communication as key determinants of fruitful collaborations [24–26]. Similar to the survey findings, trust towards the pharmacists was highlighted in interviews, where GPs and other health professionals reported that trust was developed based on pharmacists’ characteristics, competency, and performance. For pharmacists, trust appeared to be conferred on the acceptance of their recommendations or contributions by GPs and other health professionals. Trust could influence professional working relationships and commitment to collaboration between the pharmacist and other general practice team members. This finding is supported by studies that reported trust between pharmacists and GPs [27–29]. A trusting working environment can result in stronger and effective teams where employees can provide better outcomes for patients [28, 30, 31].

This study identified barriers to collaboration between the pharmacist and other health professionals in general practice teams [26, 32–34]. As general practices have busy schedules, most professionals did not have quality time to interact with the pharmacists and they had relatively limited awareness of the activities that pharmacists could perform in general practice [35]. Furthermore, interviews and survey findings highlighted that the pharmacists were more active than the GPs in contributing to relationship initiation. In addition, the absence of a role description for pharmacists hindered the initiation of professional working relationships. Role specification for general practice pharmacists could influence the establishment of trust and better utilisation of pharmacists [36]. Thus, introducing a clear role description for general practice pharmacists may improve professional working relationships, thereby enhance collaborative patient care and team effectiveness in general practice.

### Limitations

This study is subject to some limitations. The study participants were limited to eight general practices in one Australian territory. The general practices may not be representative; they displayed a high willingness to employ and collaborate with a pharmacist and had high pre-existing levels of interprofessional collaboration. Furthermore, there may have been response bias in the surveys and selection bias in the interviews. However, a purposeful sample of participants from general practice teams was utilised for the interviews to ensure a variation in the disciplines and obtain multiple perspectives.

## Conclusion

Overall, the study revealed that trust towards pharmacists was integral to professional working relationships and commitment to collaborative care in general practice teams. Interprofessional collaboration scores did not change significantly over the study, and team effectiveness of the general practice staff members was high. Introducing a clear job description for pharmacists, improving awareness of what pharmacists can do in general practice, and providing dedicated time to interact with pharmacists could be beneficial to address the barriers to collaboration, thereby improving trust and professional working relationships.

## Supplementary Information

**Additional file 1.** Survey to assess interprofessional collaboration.

**Additional file 2.** Team effectiveness survey.

**Additional file 3.** Semi-structured interview guide.

## Data Availability

All data generated or analysed during this study are included in this published article and its additional information files.
