# Supplementary material for: Interprofessional collaboration within general practice teams following the inclusion of non-dispensing pharmacists
Source: J Pharm Policy Pract. 2023 Mar 21;16:49. doi: 10.1186/s40545-023-00550-3 (PMC10031930; doi:10.1186/s40545-023-00550-3)
Supplement: Supplementary file 1 — Additional file 1. Survey to assess interprofessional collaboration. [file 40545_2023_550_MOESM1_ESM.pdf]

Additional file 1- Survey to assess interprofessional collaboration

Collaborative care survey for general practitioners

Part 1 - Demographic details

1. What is your age (Years)?

☐ 20 - 30 ☐ 31 – 40 ☐ 41 – 50 ☐ 51 – 60 ☐ > 60

2. What is your gender?

☐ Male ☐ Female ☐ Other ☐ Prefer not to say

3. How many years of experience do you have?

☐ Less than 5 ☐ 5 – 11 ☐ 12 - 18 ☐ 19 – 25 ☐ More than 25

4. Which best describes your employment status in general practice?

☐ Part-time ☐ Full-time

5. How frequently do you contact the pharmacist in general practice?

☐ Never ☐ 1-2 times/week ☐ 3-4 times/week ☐ 5 times or more/week

6. What is the most frequent method of communication between you and the pharmacist in general practice? (Select all the options that apply to you)

☐ Face to face ☐ Telephone ☐ Electronic

☐ Other (please specify) ____

7. How long have you been working with the pharmacist in general practice?

☐ Less than 6 months ☐ 6 - 12 months

☐ 12 - 24 months ☐ More than 24 months

Part 2 - Professional interactions

How frequently did the following activities occur in the LAST MONTH?

I contacted the pharmacist to discuss a patient's drug-related problem(s)

☐ Nil ☐ 1 - 2 times ☐ 3-4 times ☐ 5 times or more

The pharmacist informed me about new products/services available

☐ Nil ☐ 1 - 2 times ☐ 3-4 times ☐ 5 times or more

I contacted the pharmacist for drug information

☐ Nil ☐ 1 - 2 times ☐ 3-4 times ☐ 5 times or more

The pharmacist contacted me to discuss a patient's drug-related problem(s)

☐ Nil ☐ 1 - 2 times ☐ 3-4 times ☐ 5 times or more

I referred a patient to the pharmacist

☐ Nil ☐ 1 - 2 times ☐ 3-4 times ☐ 5 times or more

Part 3 - Exchange characteristics (Relationship initiation, role specification, trustworthiness)

Please select the answer that applies to you.

I spent time trying to learn how I can help the pharmacist to provide better care

☐ Not at all ☐ To a less extent ☐ To some extent ☐ To a moderate extent ☐ To a great extent ☐ No opinion/ Not applicable

I provided information to the pharmacist about a specific patient

☐ Not at all ☐ To a less extent ☐ To some extent ☐ To a moderate extent ☐ To a great extent ☐ No opinion/ Not applicable

I showed an interest in helping the pharmacist to improve his/her practice

☐ Not at all ☐ To a less extent ☐ To some extent ☐ To a moderate extent ☐ To a great extent ☐ No opinion/ Not applicable

Please select the answer that applies to you. *(NA- "Not applicable")*

The pharmacist is a credible professional

☐ Strongly disagree ☐ Disagree ☐ Neutral ☐ Agree ☐ Strongly agree ☐ N/A

My interactions with the pharmacist are characterised by open communication of both parties

☐ Strongly disagree ☐ Disagree ☐ Neutral ☐ Agree ☐ Strongly agree ☐ N/A

I count on the pharmacist to do what he/she says

☐ Strongly disagree ☐ Disagree ☐ Neutral ☐ Agree ☐ Strongly agree ☐ N/A

I intend to keep working together with the pharmacist

☐ Strongly disagree ☐ Disagree ☐ Neutral ☐ Agree ☐ Strongly agree ☐ N/A

I trust the pharmacist's knowledge on drugs

☐ Strongly disagree ☐ Disagree ☐ Neutral ☐ Agree ☐ Strongly agree ☐ N/A

Communication between the pharmacist and I should be two-way

☐ Strongly disagree ☐ Disagree ☐ Neutral ☐ Agree ☐ Strongly agree ☐ N/A

The pharmacist should depend on me as much as I depend on him/her

☐ Strongly disagree ☐ Disagree ☐ Neutral ☐ Agree ☐ Strongly agree ☐ N/A

I work with the pharmacist to overcome disagreements on his/her role in managing drug therapy

☐ Strongly disagree ☐ Disagree ☐ Neutral ☐ Agree ☐ Strongly agree ☐ N/A

The pharmacist and I should be mutually dependent on each other in caring for patients

☐ Strongly disagree ☐ Disagree ☐ Neutral ☐ Agree ☐ Strongly agree ☐ N/A

The pharmacist and I should negotiate to come to an agreement on the activities in managing drug therapy

☐ Strongly disagree ☐ Disagree ☐ Neutral ☐ Agree ☐ Strongly agree ☐ N/A

Part 4 – Commitment to collaboration

Please select the answer that applies to you. *(N/A- "Not applicable")*

There is cooperation between the pharmacist and my-self in managing drug therapy of our patients

☐ Strongly disagree ☐ Disagree ☐ Neutral ☐ Agree ☐ Strongly agree ☐ N/A

In making decisions for our patients, both mine and the pharmacist's options are considered

☐ Strongly disagree ☐ Disagree ☐ Neutral ☐ Agree ☐ Strongly agree ☐ N/A

Decision making is coordinated between the pharmacist and me

☐ Strongly disagree ☐ Disagree ☐ Neutral ☐ Agree ☐ Strongly agree ☐ N/A

Decision making responsibilities for the patient's drug therapy are shared between the pharmacist and myself only whenever necessary

☐ Strongly disagree ☐ Disagree ☐ Neutral ☐ Agree ☐ Strongly agree ☐ N/A

If you have any other comments or suggestions regarding collaborative care, please write in the provided space.

____

____

____

____

____

____

____

Thank you for your time and interest in completing the survey.

Collaborative care survey for other healthcare professionals in general practice

Part 1 - Demographic details

For each question below, write in the answer or select the answer applies to you.

1. What is your profession?

☐ Nurse practitioner ☐ Nurse ☐ Psychologist ☐ Dietitian

☐ Other (please specify) ____

2. What is your age (Years)?

☐ 20 - 30 ☐ 31 – 40 ☐ 41 – 50 ☐ 51 – 60 ☐ > 60

3. What is your gender?

☐ Male ☐ Female ☐ Other ☐ Prefer not to say

4. How many years of experience do you have?

☐ Less than 5 ☐ 5 – 11 ☐ 12 – 18 ☐ 19 – 25 ☐ More than 25

5. Which best describes your employment status in general practice?

☐ Part-time ☐ Full-time

6. How frequently do you contact the pharmacist in general practice?

☐ Never ☐ 1-2 times/week ☐ 3-4 times/week ☐ 5 times or more/week

7. What is the most frequent method of communication between you and the pharmacist in general practice? (Select all the options that apply to you)

☐ Face to face ☐ Telephone ☐ Electronic

☐ Other (please specify) ____

8. How long have you been working with the pharmacist in general practice?

☐ Less than 6 months ☐ 6 - 12 months

☐ 12 - 24 months ☐ More than 24 months

Part 2 - Professional interactions

How frequently did the following activities occur in the LAST MONTH?

I contacted the pharmacist to discuss a patient's drug-related problem(s)

☐ Nil ☐ 1 - 2 times ☐ 3-4 times ☐ 5 times or more

The pharmacist informed me about new products/services available

☐ Nil ☐ 1 - 2 times ☐ 3-4 times ☐ 5 times or more

I contacted the pharmacist for drug information

☐ Nil ☐ 1 - 2 times ☐ 3-4 times ☐ 5 times or more

The pharmacist contacted me to discuss a patient's drug-related problem(s)

☐ Nil ☐ 1 - 2 times ☐ 3-4 times ☐ 5 times or more

I referred a patient to the pharmacist

☐ Nil ☐ 1 - 2 times ☐ 3-4 times ☐ 5 times or more

Part 3 - Exchange characteristics (Relationship initiation, role specification, trustworthiness)

Please select the answer that applies to you.

I spent time trying to learn how I can help the pharmacist to provide better care

☐ Not at all ☐ To a less extent ☐ To some extent ☐ To a moderate extent ☐ To a great extent ☐ No opinion/ Not applicable

I provided information to the pharmacist about a specific patient

☐ Not at all ☐ To a less extent ☐ To some extent ☐ To a moderate extent ☐ To a great extent ☐ No opinion/ Not applicable

I showed an interest in helping the pharmacist to improve his/her practice

☐ Not at all ☐ To a less extent ☐ To some extent ☐ To a moderate extent ☐ To a great extent ☐ No opinion/ Not applicable

Please select the answer that applies to you. *(N/A- "Not applicable")*

The pharmacist is a credible professional

☐ Strongly disagree ☐ Disagree ☐ Neutral ☐ Agree ☐ Strongly agree ☐ N/A

My interactions with the pharmacist are characterised by open communication of both parties

☐ Strongly disagree ☐ Disagree ☐ Neutral ☐ Agree ☐ Strongly agree ☐ N/A

I count on the pharmacist to do what he/she says

☐ Strongly disagree ☐ Disagree ☐ Neutral ☐ Agree ☐ Strongly agree ☐ N/A

I intend to keep working together with the pharmacist

☐ Strongly disagree ☐ Disagree ☐ Neutral ☐ Agree ☐ Strongly agree ☐ N/A

I trust the pharmacist's knowledge on drugs

☐ Strongly disagree ☐ Disagree ☐ Neutral ☐ Agree ☐ Strongly agree ☐ N/A

Communication between the pharmacist and I should be two-way

☐ Strongly disagree ☐ Disagree ☐ Neutral ☐ Agree ☐ Strongly agree ☐ N/A

In providing patient care, I need the pharmacist as much as the pharmacist needs me

☐ Strongly disagree ☐ Disagree ☐ Neutral ☐ Agree ☐ Strongly agree ☐ N/A

The pharmacist and I mutually work with each other in caring for patients

☐ Strongly disagree ☐ Disagree ☐ Neutral ☐ Agree ☐ Strongly agree ☐ N/A

Part 4 – Commitment to collaboration

Please select the answer that applies to you. *(NA- "Not applicable")*

There is cooperation between the pharmacist and my-self in managing drug therapy of our patients when applicable

☐ Strongly disagree ☐ Disagree ☐ Neutral ☐ Agree ☐ Strongly agree ☐ N/A

There is a cooperation between the pharmacist and my-self in caring of our patients

☐ Strongly disagree ☐ Disagree ☐ Neutral ☐ Agree ☐ Strongly agree ☐ N/A

If you have any other comments or suggestions regarding collaborative care, please write in the provided space.

____

____

____

____

____

____

____

Thank you for your time and interest in completing the survey.

Collaborative care survey for pharmacists in general practice

Part 1 - Demographic details

For each question below, write in the answer or select the answer applies to you.

1 What is your age (Years)?

○ 20 – 30 ○ 31 – 40 ○ 41 – 50 ○ 51 – 60 ○> 60

2 What is your gender?

○ Male ○ Female ○ Other ○ Prefer not to say

3 How many years of experience do you have as a pharmacist?

○ Less than 5 ○ 5 – 11 ○ 12 - 18 ○ 19 – 25 ○ More than 25

4 What is your previous working background prior to commencing your career as a practice pharmacist?

○ Hospital-based pharmacy practice ○ Community pharmacy-based practice

○ Other (please specify) ____

5 What are your academic and professional qualifications?

____

____

6 How many general practitioners (GPs) are there in your practice(s)?

○ 1 – 4 ○ 5 – 9 ○ More than 10

7 Of the GPs that you are working with, how many do you work with closely? __________

8 How frequently do you contact the GP(s)?

○ Never ○ 1-2 times/week ○ 3-4 times/week ○ 5 times or more/week

9 What is the most frequent method of communication between you and the GP(s)? (Select all the options that apply for you)

○ Face to face ○ Telephone ○ Electronic

○ Other (please specify) ____

10 How long have you been working in general practice?

○ Less than 6 months ○ 6 - 12 months

○ 12 - 24 months ○ More than 24 months

Part 2 - Professional interaction

For each statement below, select the answer that applies to you.

12 How frequently did the following activities occur in the **LAST MONTH**?

	Nil	1 - 2 times	3-4 times	5 times or more
I contacted a GP to discuss a patient's medicine-related problem(s)	○	○	○	○
I informed a GP of new products/services available	○	○	○	○
I was contacted by a GP for medicine information	○	○	○	○
I was contacted by a GP to discuss a patient's medicine-related problem(s)	○	○	○	○
I received a referral from a GP (to review medicines/educate/assess adverse effects/provide more information of medicines)	○	○	○	○

Part 3 - Exchange characteristics (Relationship initiation, role specification, trustworthiness)

Complete this part of the survey based on your overall experience of working together with GP(s) to improve patient care.

14 Please select the answer that applies to you. (N/A- Not Applicable)

	Not at all	To a less extent	To some extent	To a moderate extent	To a great extent	No opinion/ N/A
I spent time trying to learn how I can help a GP to provide better care	○	○	○	○	○	○
I provided information to a GP about a specific patient	○	○	○	○	○	○
I showed an interest in helping a GP to improve his/her practice	○	○	○	○	○	○

15 Please select the answer that applies to you. (N/A- Not Applicable)

	Strongly disagree	Disagree	Neutral	Agree	Strongly agree	N/A
A GP is a credible practitioner	○	○	○	○	○	○
My interactions with a GP are characterised by open communication of both parties	○	○	○	○	○	○
I count on GP(s) to do what he/she says	○	○	○	○	○	○
I intend to keep working together with GP(s)	○	○	○	○	○	○
I trust the GP's drug expertise	○	○	○	○	○	○
Communication between a GP and me should be two-way	○	○	○	○	○	○
In providing patient care, I need GP(s) as much as GP(s) need(s) me	○	○	○	○	○	○
I should work with GP(s) to overcome disagreements on my role in managing medicines	○	○	○	○	○	○
A GP and I should be mutually dependent on each other in caring for patients	○	○	○	○	○	○
A GP and I should negotiate to come to an agreement on my activities in managing medicines	○	○	○	○	○	○

Part 4 – Commitment to collaboration

Complete this part of the survey based on your overall experience of working together with GP(s) to improve patient care.

16 Please select the answer that applies to you. (N/A- Not Applicable)

	Strongly disagree	Disagree	Neutral	Agree	Strongly agree	N/A
There is cooperation between a GP and pharmacist in managing medicines of our patients	○	○	○	○	○	○
Both GP's and pharmacist's options are considered in making decisions for our patients	○	○	○	○	○	○
Decision making is coordinated between a GP and pharmacist	○	○	○	○	○	○
Decision making responsibilities for the patient's medicines are shared between a GP and pharmacist, whenever necessary	○	○	○	○	○	○

17 If you have any other comments or suggestions regarding collaborative care, please write in the provided space.

____

____

____

Thank you for your time and interest in completing the survey.
