# Supplementary material for: Interprofessional collaboration within general practice teams following the inclusion of non-dispensing pharmacists
Source: J Pharm Policy Pract. 2023 Mar 21;16:49. doi: 10.1186/s40545-023-00550-3 (PMC10031930; doi:10.1186/s40545-023-00550-3)
Supplement: Supplementary file 2 — Additional file 2. Team effectiveness survey. [file 40545_2023_550_MOESM2_ESM.pdf]

Additional file 2 - Team effectiveness survey

Team effectiveness in general practice (This survey will be combined with CCS at the time of distribution).

Please select the answer that applies to you. (N/A- Not Applicable)

1. Membership of my team changes so frequently that we don't really have a team

☐ Strongly disagree ☐ Disagree ☐ Neutral ☐ Agree ☐ Strongly agree ☐ N/A

2. My team has the right “mix” of members—a group of people who bring different clinical perspectives and experiences to the work

☐ Strongly disagree ☐ Disagree ☐ Neutral ☐ Agree ☐ Strongly agree ☐ N/A

3. It is clear to my team what behaviour is acceptable / not acceptable

☐ Strongly disagree ☐ Disagree ☐ Neutral ☐ Agree ☐ Strongly agree ☐ N/A

4. Our practice recognises and reinforces teams that perform well

☐ Strongly disagree ☐ Disagree ☐ Neutral ☐ Agree ☐ Strongly agree ☐ N/A

5. My team has goals that are clear, useful, and appropriate to my practice

☐ Strongly disagree ☐ Disagree ☐ Neutral ☐ Agree ☐ Strongly agree ☐ N/A

6. There is a desire among team members to work collaboratively

☐ Strongly disagree ☐ Disagree ☐ Neutral ☐ Agree ☐ Strongly agree ☐ N/A

7. If asked, I could explain every team member's role and how they overlap

☐ Strongly disagree ☐ Disagree ☐ Neutral ☐ Agree ☐ Strongly agree ☐ N/A

8. My team encourages patients to be active participants in decisions about their care

☐ Strongly disagree ☐ Disagree ☐ Neutral ☐ Agree ☐ Strongly agree ☐ N/A

9. My team does a good job of helping patients understand their care plan

☐ Strongly disagree ☐ Disagree ☐ Neutral ☐ Agree ☐ Strongly agree ☐ N/A

10. The patient's needs and preferences are treated as an essential part of my team's decisions

☐ Strongly disagree ☐ Disagree ☐ Neutral ☐ Agree ☐ Strongly agree ☐ N/A

11. Each team member shares accountability for team decisions and outcomes

☐ Strongly disagree ☐ Disagree ☐ Neutral ☐ Agree ☐ Strongly agree ☐ N/A

12. My team has developed effective strategies for sharing patient treatment goals among team members

☐ Strongly disagree ☐ Disagree ☐ Neutral ☐ Agree ☐ Strongly agree ☐ N/A

13. Relevant information about changes in patient status or care plan is reported to the appropriate team member in a timely manner

☐ Strongly disagree ☐ Disagree ☐ Neutral ☐ Agree ☐ Strongly agree ☐ N/A

14. All team members effectively use the patient health record as a communication tool

☐ Strongly disagree ☐ Disagree ☐ Neutral ☐ Agree ☐ Strongly agree ☐ N/A

15. My team addresses patients' concerns effectively through team meetings and discussions

☐ Strongly disagree ☐ Disagree ☐ Neutral ☐ Agree ☐ Strongly agree ☐ N/A

16. Team meetings provide an open, comfortable, safe place to discuss concerns

☐ Strongly disagree ☐ Disagree ☐ Neutral ☐ Agree ☐ Strongly agree ☐ N/A

17. My team has an effective process for conflict management

☐ Strongly disagree ☐ Disagree ☐ Neutral ☐ Agree ☐ Strongly agree ☐ N/A

18. Overall, members of our team do a very good job of coordinating their different patient-related jobs and activities

☐ Strongly disagree ☐ Disagree ☐ Neutral ☐ Agree ☐ Strongly agree ☐ N/A

19. Members of my team act upon the information I communicate to them

☐ Strongly disagree ☐ Disagree ☐ Neutral ☐ Agree ☐ Strongly agree ☐ N/A

20. The way my team members interact makes the delivery of care highly efficient

☐ Strongly disagree ☐ Disagree ☐ Neutral ☐ Agree ☐ Strongly agree ☐ N/A

21. The way my team members interact is very good for the quality of patient care

☐ Strongly disagree ☐ Disagree ☐ Neutral ☐ Agree ☐ Strongly agree ☐ N/A

22. Working on a team like mine keeps members of my team enthusiastic and interested in their jobs

☐ Strongly disagree ☐ Disagree ☐ Neutral ☐ Agree ☐ Strongly agree ☐ N/A

23. I feel integral to my team

☐ Strongly disagree ☐ Disagree ☐ Neutral ☐ Agree ☐ Strongly agree ☐ N/A

24. I experience excellent teamwork with the members of my team

☐ Strongly disagree ☐ Disagree ☐ Neutral ☐ Agree ☐ Strongly agree ☐ N/A
