# Supplementary material for: Interprofessional collaboration within general practice teams following the inclusion of non-dispensing pharmacists
Source: J Pharm Policy Pract. 2023 Mar 21;16:49. doi: 10.1186/s40545-023-00550-3 (PMC10031930; doi:10.1186/s40545-023-00550-3)
Supplement: Supplementary file 3 — Additional file 3. Semi-structured interview guide. [file 40545_2023_550_MOESM3_ESM.pdf]

Additional file 3 - Semi-structured interview guide

- Introduction
- Permission to tape/ any questions
- Ice breaker – What is your profession? How long have you been working in this practice?

Domain	Participants	Question
Role clarity	Pharmacists	What are your roles within this practice?
		Can you describe your role? What has been the most satisfying aspect of your role in general practice?
		Describe any benefits to patients of your role?
		Describe any benefits to other healthcare professionals?
		What qualities do you need in your role (s)? Why?
		What skills do you need in your role(s)? Why?
		What CPD have you undertaken since working in general practice? Why?
		What CPD would be useful? Why?
		What other healthcare professionals work in your practice? What do you understand by their roles?
	GPs and other HCPs	What are your roles within this practice?
		Can you describe your role?
		Can you describe the practice pharmacist's role? Describe the effect that the general practice pharmacist has had on your workload? What do you think has been the effect of employing a practice pharmacist on the patients?
		What qualities do you think practice pharmacists need in their role? Why?
		What skills do you think practice pharmacists need in their role? Why?
		What CPD would you suggest for practice pharmacists? Why?
Professional interactions and trust	Pharmacists	What are your experiences in working with other HCPs in the practice?
		Who is identifying patient's medication-related problems within the practice? Can you describe examples of how a patient's medication-related problems are resolved within the practice?
		Can you describe your working relationship with GPs? Can you give examples of how you have interacted with them? What initiatives or activities have you done to establish collaboration with GPs? What worked? What did not?
		Can you describe your working relationship with other healthcare professionals? Can you give examples of how you have interacted with them? What initiatives or activities have you done to establish collaboration with other healthcare professionals? What worked? What did not?
		How much do you trust the work, decisions, views of GPs and other healthcare professionals? Have you experienced a situation

		where you feel your work or recommendation wasn't trusted by GP or other healthcare professional? How did that feel to you? How do you build trust with GPs and other healthcare professionals?<br>Can you explain communication process between you and GPs within the practice? How often during a usual week do you communicate with GPs? Has it always been this frequent? What is the method of communication? Has this changed?<br>How often during a usual week do you communicate with other healthcare professionals? Has it always been this frequent? What is the mode of communication? Has this changed?<br>Do you think that open communication occurs within your practice? Can you give some examples to support your answer to the previous question? Do you find it easy to communicate with GPs? Are there any barriers to communicating with your team? How do other team members encourage you to contribute to patient management during discussions?
	GPs and other HCPs	What are your experiences in working with practice pharmacist?<br>Can you describe examples of how a patient's medication-related problems are identified and resolved within the practice?<br>Can you describe your working relationship with practice pharmacist? Can you give examples of how you have interacted with the practice pharmacist? What initiatives or activities have you done to establish collaboration with practice pharmacist? What worked? What did not? Has this changed over time?<br>How much do you trust the work, decisions and views of the practice pharmacist? Have you experienced a situation where you feel your work or recommendation wasn't trusted by the practice pharmacist? How did that feel to you? How do you build trust with the practice pharmacist?<br>Can you explain communication process among health care professionals within the practice? How often during a usual week do you communicate with practice pharmacist? Has it always been this frequent? What is the mode of communication?<br>Do you find it easy to communicate with the pharmacist? Are there any barriers to communicating with the pharmacist? How do you encourage the pharmacist to contribute to patient management during discussions?
Collaboration and team effectiveness	Pharmacists GPs and other HCPs	What do you understand by interprofessional collaboration?<br>What were your initial thoughts on working with a team in general practice? Have they changed?<br>What is your overall view on collaborative care in general practice?

	What do you think is the impact of collaborative care on team effectiveness?<br>What are the barriers for collaboration between the practice pharmacist and GPs or healthcare professionals in general practice?<br>What are your suggestions to improve collaborative care involving the practice pharmacist in general practice?

*GP-General practitioner, HCP-Health care professional

- Is there anything else that you would like to tell me about your experiences with collaboration with other team members within general practice?
- Closing remarks
